# Influenza A Virus NS1 Protein Inhibits the NLRP3 Inflammasome

**DOI:** 10.1371/journal.pone.0126456

**Published:** 2015-05-15

**Authors:** Woo-Chang Cheong, Hye-Ri Kang, Hyunyee Yoon, Suk-Jo Kang, Jenny P.-Y. Ting, Moon Jung Song

**Affiliations:** 1 Virus-Host Interactions Laboratory, Department of Biosystems and Biotechnology, Division of Biotechnology, College of Life Sciences and Biotechnology, Korea University, Seoul, 136–713, Republic of Korea; 2 Laboratory of Protein Immunology, Biomedical Research Institute, Seoul National University Hospital, Seoul, 110–744, Republic of Korea; 3 Department of Biological Sciences, Korea Advanced Institute of Science and Technology, Daejeon, 305–701, Republic of Korea; 4 Departments of Genetics, Microbiology and Immunology, Center for Translational Immunology and Inflammatory Disease Institute, Lineberger Comprehensive Cancer Center, University of North Carolina, Chapel Hill, NC, 27599, United States of America; University of California Merced, UNITED STATES

## Abstract

The inflammasome is a molecular platform that stimulates the activation of caspase-1 and the processing of pro-interleukin (IL)-1β and pro-IL-18 for secretion. The NOD-like receptor family, pyrin domain containing 3 (NLRP3) protein is activated by diverse molecules and pathogens, leading to the formation of the NLRP3 inflammasome. Recent studies showed that the NLRP3 inflammasome mediates innate immunity against influenza A virus (IAV) infection. In this study, we investigated the function of the IAV non-structural protein 1 (NS1) in the modulation of NLRP3 inflammasome. We found that NS1 proteins derived from both highly pathogenic and low pathogenic strains efficiently decreased secretion of IL-1β and IL-18 from THP-1 cells treated with LPS and ATP. NS1 overexpression significantly impaired the transcription of proinflammatory cytokines by inhibiting transactivation of the nuclear factor-κB (NF-κB), a major transcription activator. Furthermore, NS1 physically interacted with endogenous NLRP3 and activation of the NLRP3 inflammasome was abrogated in NS1-expressing THP-1 cells. These findings suggest that NS1 downregulates NLRP3 inflammasome activation by targeting NLRP3 as well as NF-κB, leading to a reduction in the levels of inflammatory cytokines as a viral immune evasion strategy.

## Introduction

Inflammatory responses are induced in innate immune cells such as macrophages by recognizing pathogen- or damage-associated molecular patterns (PAMPs or DAMPs) by using pattern recognition receptors (PRRs). Examples of PRRs are the Toll-like receptors (TLRs), C-type lectin receptors (CLR), retinoic acid-inducible gene (RIG)-I-like receptors (RLRs), and nucleotide-binding oligomerization domain (NOD) NOD-like receptors (NLRs). Being intracellular sensors, NLRs play an important role in innate immunity and inflammation during bacterial and viral infections [1–3]. The NOD-like receptor family, pyrin domain containing 3 protein (NLRP3, also known as NALP3 or cryopyrin) is a well-characterized NLR protein, which plays a crucial role in activating inflammasome via a multiprotein complex called the NLRP3 inflammasome [4, 5]. The NLRP3 inflammasome includes the apoptosis-associated speck-like protein (ASC) and pro-caspase-1, which are involved in the secretion of interleukin (IL)-1β and IL-18 during host inflammatory responses [6–11]. NLRP3 protein recruits ASC, which in turn interacts with pro-caspase-1, leading to its cleavage and activation. Once activated, caspase-1 promotes the processing and maturation of the proinflammatory cytokines IL-1β and IL-18. These cytokines play important roles in inflammation, infiltration of immune cells, and in the secretion of other cytokines at the infection site [12].

Influenza A virus (IAV) belongs to the *Orthomyxoviridae* family, which contains negative-sense RNA as its genome. IAV infection causes respiratory diseases in humans and other animals, including birds, pigs, and horses. Depending on the strains or hosts, infection results in mild to severe illnesses [13]. In the 1918 Spain flu outbreak and the 1997 Hong-Kong flu outbreak, highly pathogenic influenza A viruses caused pandemics, leading to high mortality [14, 15]. The character of immune responses, especially the innate immune response, is considered a critical determinant of influenza outcome, as innate immunity serves as the essential first-line defense against IAV infection. NLRP3 plays an essential role in directing protective inflammatory responses that decrease mortality and limiting the overall pathogenesis of IAV infection [16–20]. M2 and PB1-F2 proteins of IAV were found to induce NLRP3 inflammasome activation [16, 18].

The genome segment 8 of the IAV encodes nonstructural protein 1 (NS1) and also produces the nuclear export protein (NEP, also called NS2) by alternatively splicing of mRNA [21, 22]. NS1 is not essential for virus replication but plays a key role as a virulence factor during infection, since it can modulate host immune responses as well as cellular functions. For example, NS1 inhibits the type I interferon (IFN) response in infected cells by targeting the activation of RIG-I [23], nuclear factor-κB (NF-κB) [24, 25], DC [26], and even adaptive immunity [21]. NS1-deleted-IAV infection showed a higher level of caspase-1 activation and IL-1β and IL-18 secretion than did wild-type IAV infection, suggesting the role of NS1 in the inflammasome [19, 27]. In this report, we studied the function of NS1 in modulating the NLRP3 inflammasome. NS1 proteins derived from a highly pathogenic strain and a low pathogenic strain of IAV were compared for their ability to modulate innate immune responses. To study the effect of NS1 in the NLRP3 inflammasome activation, THP-1, a human leukemia monocytic cell line was used because of its similarities in their characteristics compared to native monocyte derived macrophage cell [28, 29]. THP-1 cells stably expressing the NS1 proteins were generated using a lentiviral transduction system and were used to assess the function of NS1 in modulating the NLRP3 inflammasome. Our results indicated that NS1 efficiently inhibited IL-1β and IL-18 secretion from human macrophage cells, regardless of the type of strain. While the inhibition of NF-κB activation by NS1 contributed to decreased secretion of IL-1β and IL-18, NS1 exhibited physical interaction with NLRP3 and abrogated the processing of pro-IL-1β. Taken together, our results suggested that NS1 downregulated the NLRP3 inflammasome by targeting NF-κB and NLRP3, which may benefit *in vivo* virus infection by acting as an immune evasion strategy.

## Materials and Methods

### Cell culture and virus

HEK293T cells were cultured in complete Dulbecco’s modified Eagle’s medium containing 10% fetal bovine serum (FBS, HyClone, Logan, Utah, USA), supplemented with 100 units/mL penicillin and 100 μg/mL streptomycin (HyClone, Logan, Utah, USA). THP-1 cells, human leukemia monocytic cells were cultured in RPMI1640 medium, supplemented with 10% FBS, 10 mM HEPES, 100 units/mL penicillin, 100 μg/mL streptomycin (HyClone, Logan, Utah, USA), and 50 μM beta-mercaptoethanol (Sigma, St. Louis, MO, USA). Sendai virus (SeV) was originally obtained from Dr. Peter Palese (Icahn School of Medicine at Mount Sinai, USA) and propagated in 10-day-old embryonated eggs at 37°C for 48 h. After chilling at 4°C overnight, the titer of amplified SeV was determined using hemagglutinin assays. The viruses were stored in aliquots at liquid nitrogen until use.

### Plasmids

NS1-WSN constructs were prepared from a pHW-2000-NS construct [30]. NS1-HK was synthesized by GenScript (GenScript Corporation, Piscataway, NJ; www.genscript.com) by applying codon optimization for humans. Each NS1 variant construct was cloned into a pENTR vector of the Gateway system (Invitrogen, Carlsbad, CA, USA) by using the following primers: NS1-HK, F-5’-CGACAGCCAGCGAATTCATGAATAGCAATACTGTG-3’ and R-5’- GCATATGCGGCCGCTCACACTTCAGGTTCAATAG-3’ and NS1-WSN, F-5’-CCGGAATTCATGGATCCAAACACTGTGTCAAGC-3’ and R-5’- GCGGCCGCTCAAACTTCTGACCTAATTGTTCCC-3’. NS1-WSN Δ40–80 construct was generated by a two-step PCR approach. NS1-WSN (1–39) sequence was amplified by primers: F-5’-CCGGAATTCATGGATCCAAACACTGTGTCAAGC-3’ and R-5’-GCCATTGATCTCGGCCGAAGCCGATCAAGGAATGG-3’. NS1-WSN (81–230) sequence was amplified by primers: F-5’-GATCAATGGCCTCTGTACCTGCATCGCGCTACCTAACTG-3’ and R-5’- GCGGCCGCTCAAACTTCTGACCTAATTGTTCCC-3’. In the next PCR step, two PCR products were mixed as templates and amplified by primers: F-5’-CCGGAATTCATGGATCCAAACACTGTGTCAAGC-3’ and R-5’- GCGGCCGCTCAAACTTCTGACCTAATTGTTCCC-3’. The entry clones were further transferred to a 6×MYC-tagged (pCS3-MT-6-MYC) destination vector containing additional sequences to generate MYC-tagged NS1 variants by using the Gateway technology (Invitrogen, Carlsbad, CA, USA), according to the manufacturer’s instructions. To construct an MYC-NS1 variant-expressing lentiviral vector, fragments of MYC-NS1 variants were PCR-amplified from pCS3-MT-6-MYC-NS1 variant constructs by using the following primers: MYC-NS1-HK, F-5’-CTAGCTAGCATGGAGCAAAAGCTCATTTCTG-3’ and R-5’-GCATATGCGGCCGCCACTTCAGGTTCAATAG-3’, MYC-NS1-WSN and MYC-NS1-WSN Δ40–80, F-5’-CTAGCTAGCATG GAGCAAAAGCTCATTTCTG-3’ and R-5’-GATATATGCGGCCGCAACTTCTGACCTAATTGTTCC-3’, and MYC-tag alone as control, F-5’-GCGGACGCTAGCTAGCATGGAGCAAAAGCTCATTTC-3’ and R-5’-GTATAGATATGCGGCCGCGAATTCGGTACCGGATCC-3’. The amplified fragments were then cloned into modified lentiviral pCDH-MCS-T2A-copGFP-MSCV (System Biosciences, Mountain View, CA), as described previously [31] by using *Nhe*I and *Not*I sites.

### Transfection and Transduction

Polyethylenimine (1 mg/mL) (Sigma, St. Louis, MO, USA) was used for the transfection of HEK293T cells, as previously described [32]. To obtain NS1-expressing lentiviruses, each NS1-containing lentiviral vector and packaging vector (pMD2.G and psPAX2, from Dr. Seungmin Hwang, The University of Chicago, IL, USA) were co-transfected into HEK293T cells. For transduction, the supernatants were then incubated with THP-1 cells, and the medium was replenished every 24 hr for 3 days. The control THP-1 cells were transfected with a lentiviral vector expressing MYC-tag alone.

### Luciferase reporter assays

The luciferase reporter assay system (Promega, Medison, USA) was used to measure the promoter activity (IFN-β-luc and 2xκB-luc). To test IFN-β promoter activity, HEK293T cells were transfected with reporter constructs (IFN-β-luc) and NS1 expression plasmids. After 24 hours post-transfection, the cells were infected with SeV (20 HA/well). After 1 h of adoption, the cells were washed with phosphate-buffered saline (PBS) and added to fresh medium. After 16 hours incubation, the cells were harvested and analyzed for the luciferase reporter assays, according to manufacturer’s instructions. To test 2xκB promoter activity, HEK293T cells were transfected with reporter constructs (2xκB-luc) and NS1 expression plasmids in the presence of a p65-expressing plasmid. After 26 hours post-transfection, the cells were harvested and analyzed by the luciferase reporter assay. Each transfection was performed in triplicate, with β-galactosidase-expressing plasmid as an internal control, while a vector expressing MYC-tag alone was used as a control vector. In all assays, luminescence from the reporters was normalized to the activity of β-galactosidase.

### ELISA

THP-1 cells expressing NS1 variants were differentiated with 12-O-tetradecanoylphorbol-13-acetate (TPA, Sigma, St. Louis, MO, USA) (50 ng/mL) for 3 days. Differentiated THP-1 macrophage cells were treated with 1 μg/mL LPS (Sigma, St. Louis, MO, USA) for 6 hr, followed by ATP treatment (2.5 mM, Sigma, St. Louis, MO, USA) for 15 min. Concentrations of IL-1β and TNF-α in the supernatants were measured by the Bio-Plex Pro assay kits (BIO-RAD Laboratories, Inc., USA) and human IL-18 ELISA kit (MBLTM International Co., Japan), respectively, according to the manufacturer’s instructions.

### Quantitative real-time PCR

Total RNA was extracted using the Tri reagent (MRC, Cincinnati, USA) and by chloroform extraction methods for the quantification of cellular transcripts. cDNAs were synthesized using a RevertAid First strand cDNA synthesis kit (Thermo scientific, Lithuania) with random hexamers. Transcripts were quantified by using primers for the target genes pro-IL-18 (F-5’-AAACTCATGACCAGTCTGCA-3’ and R-5’-AGGAGATCTTCAGTTTCGGAGC-3’), pro-IL-1β (F-5’-GTGGCAATGAGGATGACTTGTTC-3’ and R-5’-TAGTGGTGGTCGGAGATTCGTA-3’) and TNF-α (F-5’-CTGCTGCACTTTGGAGTGAT-3’ and R-5’-AGATGATCTGACTGCCTGGG-3’) [33] and their levels were normalized to those of actin (F-5’-GTATCCTGACCCTGAAGTACC-3’ and R-5’-TGAAGGTCTCAAACATGATCT-3’). The experiment was performed on iCycler iQ multicolor Real-time PCR detection system and analyzed on Optical Bio system software (Bio-Rad). SYBR Green PCR was run at 95°C for 15 min, followed by 55 cycles at 95°C for 30 sec, 57°C for 30 sec, and 72°C for 30 sec. PCR was followed by a melting curve analysis.

### Western blot analysis

For western blot analysis, cells were harvested in a buffer containing 62.5 mM Tris-HCl (pH 6.8), 20% glycerol, 5% β-mercaptoethanol, 2% SDS, and 0.025% bromophenol blue. The whole-cell lysates were resolved by SDS-PAGE, transferred to a polyvinylidene fluoride membrane, and probed with primary antibodies against FLAG-M2 (1:2,000; Sigma, St. Louis, MO, USA), MYC (1:2000, Laboratory manufactured), IκBα (1:500; Cell Signaling, Danvers, Massachusetts, USA), p65 (1:500; Cell Signaling, Danvers, Massachusetts, USA), phospho-p65 (1:500; Cell Signaling, Danvers, Massachusetts, USA), caspase-1 (1:500; Santa Cruz Biotechnology, Texas, USA), IL-1β (1:500; Santa Cruz Biotechnology, Texas, USA), NLRP3 (1:2000; Adipogen, Schützenstrasse 12, 4410 Liestal, Switzerland, it is kindly provided by Dr. Je-Wook Yu, Yonsei University) and α-tubulin (1:2,000; Sigma, St. Louis, MO, USA). Goat anti-rabbit, mouse anti-goat, and goat anti-mouse immunoglobulin G conjugated with horseradish peroxide secondary antibody (Santa Cruz Biotechnology, Texas, USA) was detected by ECL plus western blotting detection reagents (ELPIS, Taejeon, Korea), and the signals were detected and analyzed using LAS-4000, a chemiluminescent image analyzer (Fujifilm).

### Immunoprecipitation assay

HEK293T cells were seeded, transfected with the indicated plasmids, and incubated for 48 hr. THP-1 cells expressing NS1 variants were differentiated with TPA for 3 days. Differentiated THP-1 macrophage cells were treated with 1 μg/mL LPS for 6 hr, followed by ATP treatment. Cells were scraped and resuspended in the IP buffer (20 mM HEPES (pH 7.4), 100 mM NaCl, 0.5% Nonidet P-40, and 1% Triton X-100), supplemented with 1/100 volume of protease inhibitor cocktail (Sigma, St. Louis, MO, USA). Cell lysates were rotated at 4°C for 1 hr, and cell debris was removed by centrifugation (13,000 g, 4°C, 10 min). FLAG-M2 antibodies (Sigma, St. Louis, MO, USA) or MYC antibodies were added and the lysates were incubated at 4°C with rotation. Thereafter, 30 μL protein A/G agarose beads (Pierce, Rockford, IL, USA) were added and incubation was continued for 16 hr at 4°C. The beads were washed extensively using IP buffer, and the proteins were analyzed by western blot analysis.

### Immunofluorescence assay (IFA) and confocal microscopy

HEK293T cells were seeded onto a cover glass in a 24-well plate. On the following day, HEK293T cells were transfected with the indicated plasmids. After 24 hr, cells were fixed for 15 min with 4% paraformaldehyde and 0.15% picric acid in PBS. The blocking step was performed with 10% normal goat serum with 1× PBS containing 0.3% Triton X-100 and 0.1% BSA. Anti-MYC was used as the primary antibody and was incubated with the cells for 16 hr at 4°C. Mouse-Cy3 (Jackson ImmunoResearch, West Baltimore Pike West Grove, PA, USA) was used as the secondary antibody and was incubated with the cells for 45 min at room temperature. DAPI stain (1:1,000) was used for nuclear staining for 3 min at room temperature. NS1 variant-expressing THP-1 cells were seeded on the cover glass in a 24-well plate and differentiated with TPA (50 ng/mL) treatment for 3 days. After differentiation, IFA was performed as described above. The fluorescence images were obtained at a magnification of 1000× by using a confocal laser scanning microscope (LSM 5 Exciter, Zeiss).

## Results and Discussion

### Characteristics of NS1 variants

**Fig 1:**
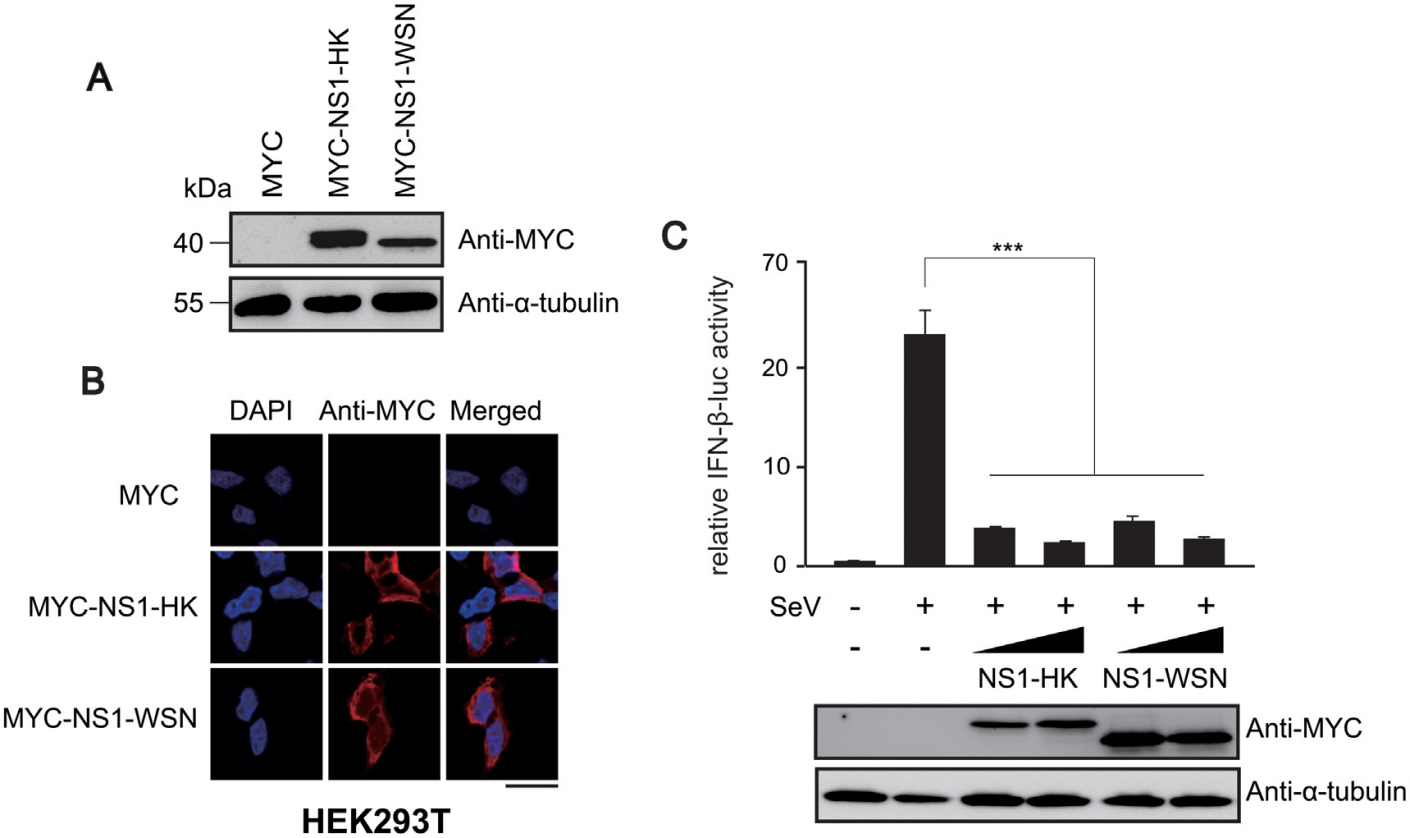
NS1 variants and their characterization. Plasmids expressing the NS1 variants NS1-HK and NS1-WSN were transiently transfected into HEK293T cells (A and B). (A) Expression of NS1 variants in the cells was examined by western blot analysis using an anti-MYC antibody. Tubulin was used as a loading control. (B) HEK293T cells expressing NS1 variants were fixed at 24 hr post-transfection, immunostained with anti-MYC (red) for NS1 detection, and examined under a confocal laser scanning microscope. Nuclei were stained with DAPI (blue). Scale bar, 20 μm. (C) Transfection of HEK293T cells was performed in triplicate by using the reporter construct (IFN-β-luc) and the NS1 expression plasmids (300 and 500 ng); the β-galactosidase expression plasmid served as an internal control. At 24 hr post-transfection, 20 HA of SeV was infected. After 16 hr infection, IFN-β-luc reporter activities were measured and normalized to β-galactosidase activities. Statistical analysis was performed using Student’s *t*-test (*** denotes a *p*-value of <0.005.).

Many variants of NS1 proteins exist, depending on the strain of the influenza A virus. Although the degree of variance between the NS1 variants is moderate and their biological functions are basically identical, some studies have reported differences in their properties [34, 35]. We performed protein sequence alignment of NS1 variants derived from highly pathogenic viruses such as A/Hong Kong/483/1997(H5N1) and A/Brevig Mission 1/1918(H1N1), as well as low pathogenic viruses such as A/PR/8/34 (H1N1) and A/WSN/1933 (H1N1) by using Clustal Omega software (http://www.ebi.ac.uk/Tools/msa/clustalo/) (S1 Fig). NS1 variants showed up to 85.2% identity in their amino acid sequences. While the sequence identity of NS1 was quite high among H1N1 strains (93.9%), sequence alignment of the NS1 variants revealed six amino acid residues (aa 3, 22, 91, 114, 215, and 224) that are conserved within highly pathogenic strains, but not among low pathogenic strains (S1B Fig). The domains and residues important for NS1 specific functions are largely identical among different strains [36]: double stranded RNA-binding (R38 and R41) [36] and TRIM25-mediated antiviral IFN-response (E96 and E97) [23]. In contrast, NS1 binding residues (aa 103, 106 and 189) for cleavage and polyadenylation specificity factor (CPSF30) [35, 37–39] and some residues in the poly(A)-binding protein II (PABPII) binding site are different among strains [40]. However the difference of these amino acids does not appear to attribute to the pathogenicity of influenza A virus as they are not conserved among highly pathogenic strains. For this study, the NS1 protein of A/HK/483/97(H5N1) (NS1-HK; a highly pathogenic strain and that of A/WSN/33(H1N1) (NS1-WSN; a low pathogenic strain) were selected, since they had the lowest identity score among the NS1 variants. Cloned MYC-tagged NS1-HK and NS1-WSN were transiently transfected into HEK293T cells, and their expressions were detected using western blot assays (Fig 1A). Immunofluorescence assays in HEK293T cells showed that MYC-tagged NS1-HK protein and NS1-WSN protein were dominantly localized in the cytoplasm (Fig 1B). To confirm the functionality of these clones, MYC-tagged NS1-HK or NS1-WSN was co-transfected with IFN-β-Luc into HEK293T cells. At 24 hour post-transfection, the cells were infected with SeV that is known to elicit the IFN- β response in a RIG-I dependent manner (Fig 1C). Consistent with the previously identified function of NS1 [41, 42], both MYC-tagged NS1-HK and NS1-WSN were able to efficiently suppress RIG-I-mediated IFN-β transactivation.

### Downregulation of NLRP3 inflammasome activity in NS1-expressing THP-1 macrophage cells

**Fig 2:**
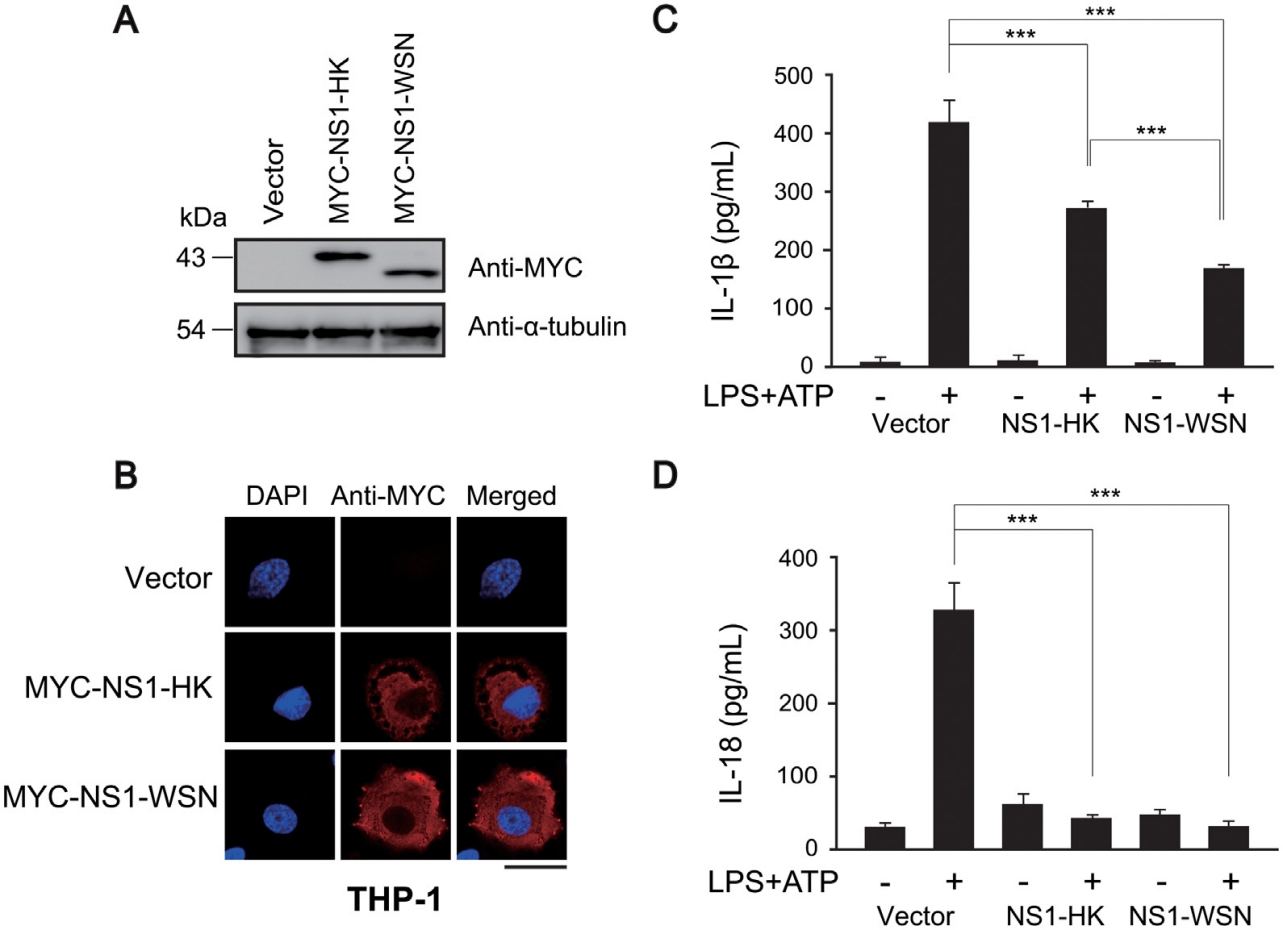
Expression of NS1 variants in THP-1 macrophage cells and their inhibitory effects on NLRP3 inflammasome. (A) NS1 variants were expressed in THP-1 cells by using a lentivirus vector transduction system. After transduction, the expression of each NS1 variant in differentiated THP-1 cells was evaluated by western blot analysis. (B) NS1 variant-transduced THP-1 macrophage cells were immunostained with anti-MYC (red) for NS1 detection. Localization was examined under a confocal laser scanning microscope. Nuclei were stained with DAPI (blue). Scale bar, 20 μm. (C and D) Transduced THP-1 macrophage cells were differentiated with TPA and then treated with LPS (1 μg/mL) for 6 hr, followed by treatment with ATP (2.5 mM) for 15 min. The supernatants were harvested and subjected to ELISA to quantify IL-1β and IL-18. Data represent the mean and standard deviation. Statistical analysis was performed using Student’s *t*-test to analyze the differences between control and NS1-expressing samples (*** denotes a *p*-value of <0.005.).

To elucidate NS1 function in the inflammasome, lentiviruses expressing NS1 variants were transduced into THP-1 macrophage cells. Expression and subcellular localization of the MYC-tagged NS1 proteins in transduced THP-1 macrophage cells were examined by western blot and immunofluorescence assays, respectively (Fig 2A and 2B). NS1 proteins in transduced THP-1 macrophage cells were expressed at the predicted molecular size (Fig 2A) and were localized dominantly in the cytoplasm, regardless of the type of strain (Fig 2B). NS1 protein is shown to possess a nuclear export signal in addition to a nuclear localization signal, thereby functioning both in the cytoplasm and in the nucleus [43]. Although nuclear localization of NS1 is frequently found, its cytoplasmic localization is not uncommon as NS1 localization can vary depending on the stains and the cell types [44]. To test the effect of NS1 on the NLRP3 inflammasome, THP-1 macrophage cells expressing MYC-tagged NS1-HK or NS1-WSN, as well as control THP-1 cells were primed with lipopolysaccharide (LPS), and then treated with ATP, a known NLRP3 agonist [6, 8, 9, 45]; the levels of secreted IL-1β and IL-18 in the supernatants were measured by ELISA (Fig 2C and 2D). Compared with the control THP-1 cells, THP-1 macrophage cells expressing either of the NS1 variants secreted lower levels of IL-1β and IL-18. These results suggest that NS1 may act as a down-regulator of the NLRP3 inflammasome, since MYC-tagged NS1-HK and NS1-WSN inhibited IL-18 and IL-1β secretion induced by activation of the NLRP3 inflammasome.

### Inhibition of the first signal of the NLRP3 inflammasome by NS1

**Fig 3:**
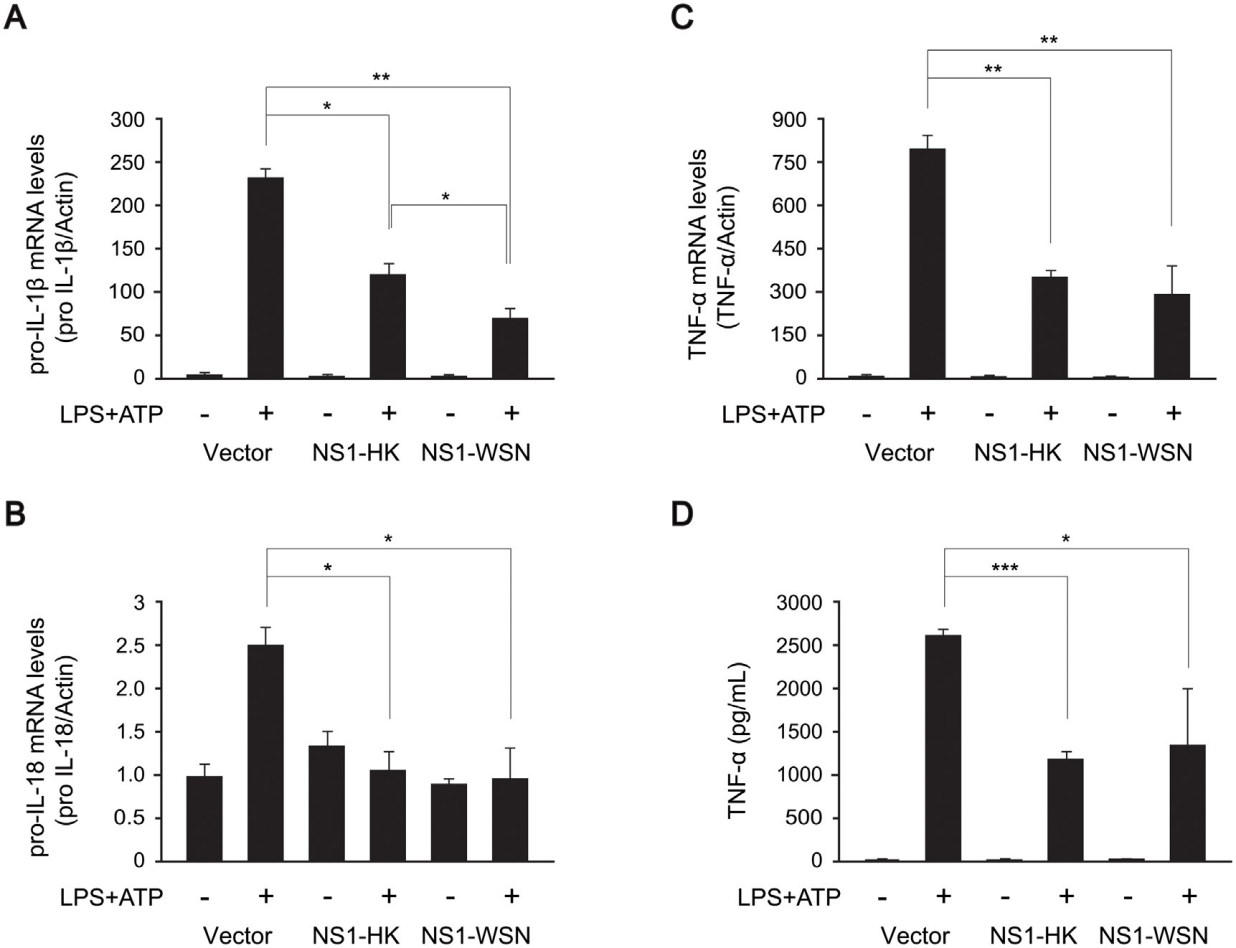
Downregulation of the transcription of proinflammatory cytokines by NS1 variants. NS1 variants-transduced THP-1 macrophage cells were differentiated with TPA and then treated with LPS (1 μg/mL) for 6 hr and subsequently with ATP (2.5 mM) for 15 min. (A-C) mRNA levels of pro-IL-1β (A), pro-IL-18 (B), and TNF-α (C) were analyzed by quantitative real-time PCR using gene-specific primers. (D) The supernatants were harvested and subjected to ELISA to quantify TNF-α. Data represent the mean and standard deviation. Statistical analysis was performed using Student’s *t*-test to analyze the differences between control and NS1-expressing samples (* denotes a *p*-value of <0.05; ** denotes a *p*-value of <0.01; *** denotes a *p*-value of <0.005).

Two distinct signals are required to induce the secretion of IL-1β and IL-18 cytokines via the NLRP3 inflammasome in macrophages [3, 8, 9, 19]. The first signal is to increase the transcription and protein synthesis of pro-IL-1β and pro-IL-18, which are mainly mediated by NF-κB activation. The second signal, also called the danger signal, is to induce the assembly of the NLRP3 inflammasome components such as NLRP3, ASC, and pro-caspase-1, which, in turn, activates caspase-1, leading to cleavage and maturation of IL-1β and IL-18 for secretion. Thus, we examined which step of the NLRP3 inflammasome activation might have been disrupted by NS1 proteins. First, we tested the effect of NS1 on transcription of pro-inflammatory cytokines. The levels of pro-IL-1β and pro-IL-18 transcripts were decreased in THP-1 cells expressing MYC-tagged NS1-HK or NS1-WSN proteins (Fig 3A and 3B). It was noted that not only the secretion, but also the transcription of IL-18 was highly suppressed by NS1, compared to those of IL-1β (Fig 2C–2D and Fig 3A and 3B). Although the reasons are not clear, it is plausible to think that NS1 might have a stronger effect on IL-18 than IL-1β at the promoter levels. Furthermore, suppression of transcription by NS1 was not limited to transcription of pro-IL-1β and pro-IL-18, as the level of tumor necrosis factor -α (TNF-α) mRNA was also significantly decreased (Fig 3C). The secreted TNF-α protein was consistently reduced in MYC-tagged NS1-expressing THP-1 macrophage cells treated with LPS and ATP (Fig 3D).

**Fig 4:**
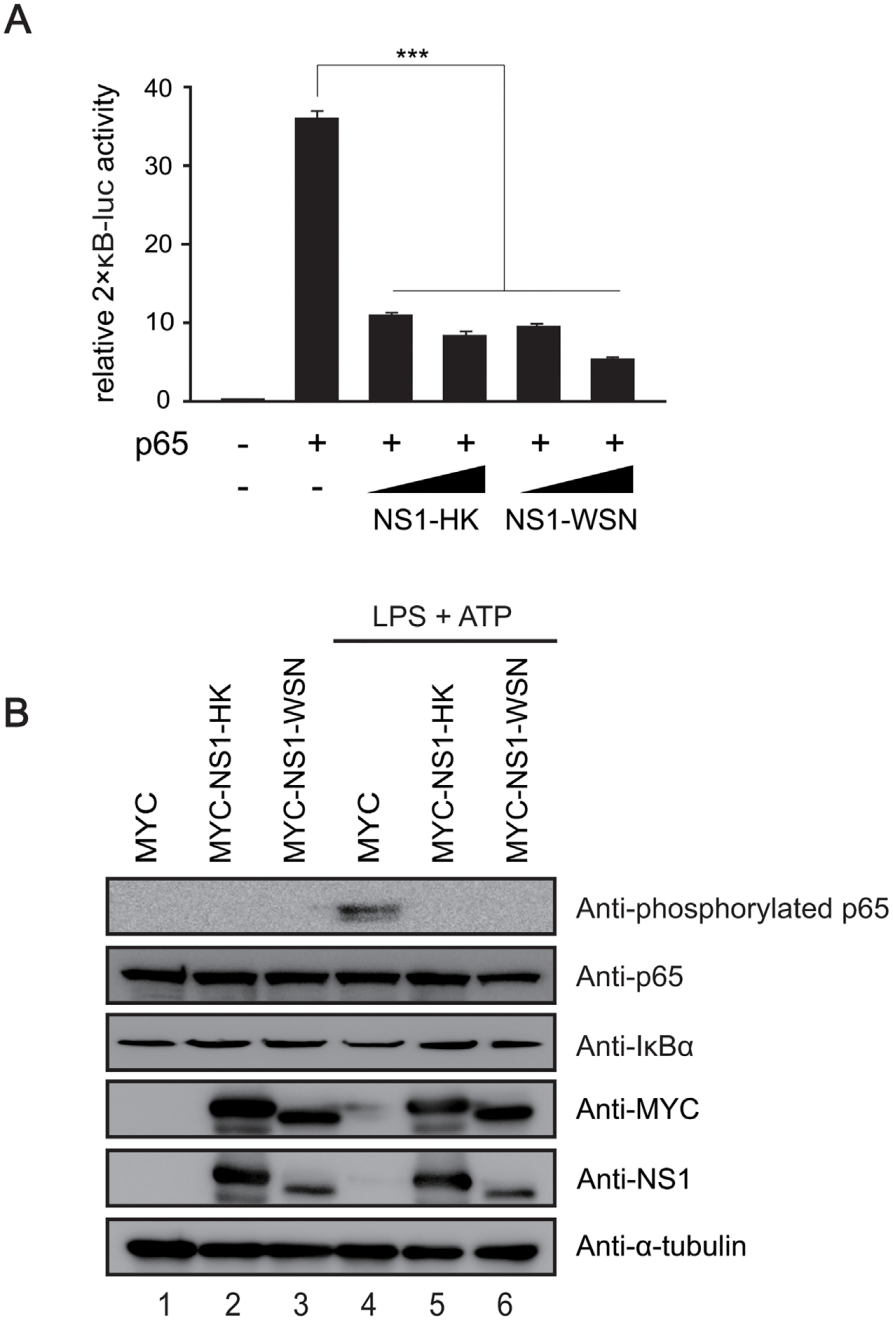
Downregulation of NF-κB activation by NS1 variants. (A) Downregulation of p65-mediated 2xκB-luc activity by NS1 variants. HEK293T cells were transfected with reporter constructs (2xκB-luc) and NS1 expression plasmids (300 and 500 ng) in the presence of p65-expressing plasmid. Each transfection was performed in triplicate, with the β-galactosidase expression plasmid serving as an internal control. After 26 hr transfection, 2xκB-luc reporter gene activities were measured and normalized to β-galactosidase activities. Statistical analysis was performed using Student’s *t*-test (*** denotes a *p*-value of <0.005). (B) Inhibition of the NF-κB pathway by NS1 variants in THP-1 macrophage cells. Transduced THP-1 cells were differentiated with TPA treatment, and then treated with LPS (1 μg/mL) for 6 hr, followed by treatment with ATP (2.5 mM) for 15 min. Each indicated proteins were identified by western blot analysis.

Next, we investigated whether the transcription of proinflammatory cytokines was downregulated by NS1 via suppression of NF-κB activation. To test the effect of NS1 on NF-κB activation, HEK293T cells were transfected with 2xκB-Luc, p65, and NS1 variants for 26 hr, and the 2xκB-Luc activity was measured. In accordance with the findings of previous studies [24, 25], NS1-HK and NS1-WSN efficiently inhibited NF-κB activation (Fig 4A). We also examined whether NS1 could downregulate the activation of NF-κB in the context of NLRP3 inflammasome activation. When treated with LPS and ATP, compared with the control THP-1 cells, THP-1 cells expressing NS1 variants exhibited a higher level of IκBα and a lower level of p65 phosphorylation (Fig 4B). Taken together, these results indicate that both NS1-HK and NS1-WSN efficiently suppress the first signal of the NLRP3 inflammasome activation by inhibiting the NF-κB activation pathway.

### Inhibition of the second signal of the NLRP3 inflammasome by NS1

**Fig 5:**
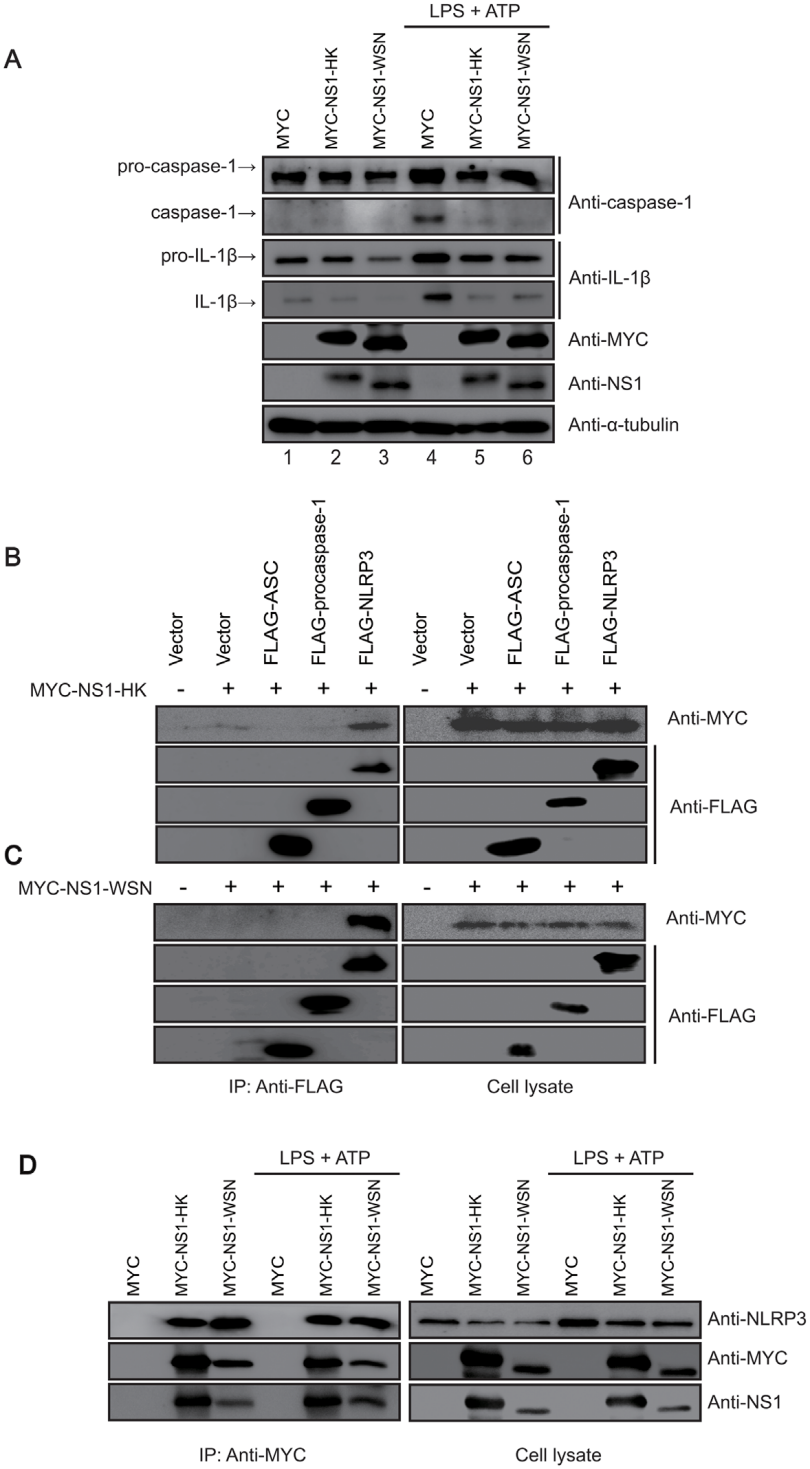
Downregulation effect of NS1 variants on the NLRP3 inflammasome and their interaction with NLRP3. (A) Differentiated THP-1 cells expressing NS1 variants were treated with LPS (1 μg/mL) for 6 hr, followed by treatment with ATP (2.5 mM) for 15 min. The cell lysates were obtained and subjected to western blot analysis using the indicated antibodies. (B and C) NS1 variants interacted with NLRP3, not with ASC or pro-caspase-1. The MYC-NS1-HK (B) or MYC-NS1-WSN (C) construct was co-transfected with a FLAG-tagged NLRP3 inflammasome protein construct (NLRP3, ASC, or pro-caspase-1) into HEK293T cells. The cells were harvested at 48 hr post transfection, and lysates were immunoprecipitated with anti-FLAG-M2 antibody. Protein interaction with NS1 was identified by western blot analysis with an anti-MYC antibody. (D) Differentiated THP-1 cells expressing NS1 variants were treated with LPS (1 μg/mL) for 6 hr, and subsequently with ATP (2.5 mM) for 15 min. The cells were harvested and lysates were immunoprecipitated with anti-MYC antibody. Endogenous NLRP3 protein interaction with NS1 was identified by western blot analysis with anti-NLRP3.

NS1 was found to modulate the first signal of NLRP3 inflammasome activation. Since targeting the first and the second signals may not be mutually exclusive, we further investigated the role of NS1 in the second signal of the NLRP3 inflammasome, which involves processing pro-IL-1β and pro-IL-18 cytokines for secretion. THP-1 cells expressing MYC-tagged NS1 variants were treated with LPS and ATP and subjected to western blot analysis using anti-IL-1β antibody. The results indicated that MYC-tagged NS1-expressing THP-1 cells barely processed pro-caspase-1 and pro-IL-1β, while the control cells processed pro-caspase-1 and pro-IL-1β efficiently, thereby forming caspase-1 and IL-1β, suggesting that NS1 may inhibit the NLRP3 inflammasome by ablating the second signal (Fig 5A). To investigate the physical involvement of NS1 in inhibition of the NLRP3 inflammasome, HEK293T cells were transiently transfected with MYC-tagged NS1 and NLRP3 inflammasome components such as NLRP3, ASC, and caspase-1, and were subjected to immunoprecipitation. The results showed that both NS1-HK and NS1-WSN bound to NLRP3, not to ASC or pro-caspase-1 (Fig 5B and 5C). Furthermore, upon transduction of THP1 cells with the lentivirus expressing NS1-HK or NS1-WSN, we found that endogenous NLRP3 interacted with NS1 proteins, regardless of their originated strains (Fig 5D). Taken together, these results suggested that NS1 may target endogenous NLRP3 to down-regulate NLRP3 inflammasome activation via physical interaction, thereby inhibiting the activation of pro-caspase-1 and the maturation of IL-18 and IL-1β.

It was reported that the NLRP3 inflammasome might play a critical role in eliciting host innate immunity that limits the overall pathogenesis of influenza A virus infection *in vivo* [16–20]. M2 and PB1-F2 viral proteins of IAV were shown to induce NLRP3 inflammasome activation, as the host inflammasome system may detect IAV infection through disturbance in the ionic environment (M2) during the early phase of infection or directly sensing viral protein (PB1-F2) upon viral gene expression [16, 18]. However, the virus certainly develops a strategy to modulate the NLRP3 inflammasome. Here, we showed that NS1 efficiently suppressed secretion of IL-1β and IL-18 cytokines by inhibiting both transcription and processing of pro-IL-1β and pro-IL-18 (Figs 4 and 5). In addition to the inhibitory effect of NS1 on NF-κB activation as shown in Fig 4, NS1 can inhibit CPSF30-mediated cellular pre-mRNA processing and export to the cytoplasm [38, 46, 47], which may further decrease the levels of pro-IL-1β and pro-caspase-1 expression in NS1-expressing THP-1 cells (Fig 5A). Furthermore, inhibition of pro-IL-1β and pro-IL-18 processing by NS1 may be attributed to the direct targeting of NLRP3. The interaction of NS1 with NLRP3 suggests that NS1 may inhibit NLRP3 inflammasome activation by interrupting NLRP3 oligomerization, interfering with NLRP3-ASC interactions, or inducing conformational changes in NLRP3, thereby rendering it malfunction. Further studies are warranted to investigate the detailed molecular mechanisms.

Our results were consistent with those of previous studies that showed that NS1 downregulates secretion of IL-1β and IL-18 by inhibiting NLRP3 inflammasome activation [19, 27]. However, our study differs from previous studies in terms of the system used to define the function of NS1. We used human macrophage cells that stably expressed MYC-tagged NS1 and induced activation of the NLRP3 inflammasome by treatment with ATP, while others utilized an IAV infection system with NS1 mutant viruses [19, 27]. Although infection system is more physiologically relevant in most studies, NS1-deleted mutants are shown to be complicated. According to a recent report, NS1-deleted mutant viruses generated by the conventional cloning technology could also result in truncation of the NEP coding region in the segment 8 genome, thereby potentially affecting the viral phenotype due to the aberrant production of NEP [48]. By selectively silencing NS1 through miRNA, Chua et al. showed that silencing NS1 had no impact on virus replication *in vitro*, whereas NS1-deleted mutant virus was defective in *in vitro* replication [48]. Thus, it is worth of examining the function of NS1 in immune cells, independent of other viral proteins to clarify this issue.

In an attempt to validate a functional domain of NS1 critical for NLRP3 inhibition, we generated a lentivirus expressing a NS1-WSNΔ40–80 construct. The NS1 domain of aa 40–80 was previously reported to be important for the regulation of the inflammasome using the infection system [19, 27]. Our results show that NS1-WSNΔ40–80 exhibited an identical phenotype as the wild type in that NS1-WSNΔ40–80 down-regulated IL-1β and IL-18 to the same levels as the wild-type and was able to inhibit caspase-1 processing as well as NF-κB activation (S2A–S2D Fig). Moreover NS1-WSNΔ40–80 strongly interacted with NLRP3 (S2E Fig). These results suggest that the domain of aa 40–80 might be dispensable for NS1 function in down-regulating the NLRP3 inflammasome. Nevertheless, discrepancy of the mutant phenotypes between infection and overexpression systems should be noted and careful examination using the recently developed knock-down system may help delineate critical domains of NS1 functions [48].

Pothlichet *et al*. reported that NS1 inhibited NLRP3 inflammasome by interfering with RIG-I that could activate the inflammasome in primary lung epithelial cells upon influenza virus infection. Instead of using viral infection, here we used a typical NLRP3 agonist to activate the NLRP3 inflammasome in human macrophages, bypassing the RIG-I signaling pathway, thereby allowing us to evaluate the role of NS1 in the NLRP3 inflammasome directly. While our results suggest similar effects of different NS1 variants on the NLRP3 inflammasome, regardless of the strain origin of the proteins used (NS1-HK from a highly pathogenic strain versus NS1-WSN from a low pathogenic strain), Pothlichet *et al*. showed that NS1 derived from a highly pathogenic strain (A/Brevig Mission/1/1918, a 1918 pandemic strain) was more potent in inhibiting the NLRP3 inflammasome *in vivo* than one from a less pathogenic strain (A/PR/8/34), as the former interacted more strongly with RIG-I in lung epithelial cells [19]. These differences could be attributed to the differences between the viral strains (the 1918 Spain pandemic strain versus the 1997 Hong Kong pandemic strain), the cell types (lung epithelial cells versus macrophages), and/or the experimental conditions (RIG-I-mediated NLRP3 inflammasome activation upon infection versus NLRP3 agonist treatment upon NS1 overexpression). Nonetheless, both studies suggest that functional inhibition of the NLRP3 inflammasome by NS1 may play an important role in ablating host immune responses.

Since NLRP3 inflammasome requires two signals for activation, suppression of the first signal could also contribute to inhibition of the NLRP3 inflammasome by NS1. Previous studies have shown that NS1 inhibited NF-κB activation by targeting IKK [24, 25]. Consistent with the results of these studies, our results indicated that p65 phosphorylation was diminished concomitantly with a higher level of IκBα in MYC-tagged NS1-expressing THP-1 macrophage cells (Fig 4). Consequently, pro-IL-1β pro-IL-18, and TNF-α were decreased at the mRNA levels as well as the protein levels in the presence of MYC-tagged NS1 variants (Figs 3 and 5A). Taken together, our results demonstrated inhibitory effects of NS1 on the NLRP3 inflammasome by targeting the two signals of the NLRP3 inflammasome, NF-κB was targeted in the first signal and NLRP3 was targeted in the second signal; this served as an effective strategy to evade host immune responses.

## Supporting Information

S1 Fig Alignment of NS1 sequences. (A) A schematic diagram of NS1 protein indicating functional domains and residues critical for its binding with dsRNA, TRIM25, CPSF30, and PABPII. (B) Sequence alignment of NS1 variants. NS1 proteins of highly pathogenic influenza A viruses such as A/Brevig Mission/1/1918 H1N1 and A/Hong Kong/483/1997 H5N1 (blue letters) and those of low pathogenic influenza A viruses such as A/Puerto Rico/8/34 H1N1 and A/WSN/1933 H1N1 (black letters) were aligned by the ‘Clustal Omega’ multiple sequence alignment program. Black boxes indicate six amino acid residues that were conserved among highly pathogenic strains, but not among low pathogenic strains. Gray boxes indicate residues that are different between the H1N1 strains and the H5N1 strain. White boxes indicate residues that are different, but non-characteristic for pathogenic strains. Every tenth amino acids are marked by bars.
(PDF)

S2 Fig Effects of NS1-WSN Δ40–80 on the NLRP3 inflammasome. THP1 cells were transduced with lentiviruses expressing MYC-tag alone, MYC-NS1-WSN, or MYC-NS1-WSN Δ40–80, and then differentiated with TPA. Differentiated THP-1 cells expressing NS1-WSN or NS1-WSN Δ40–80 were, then, treated with LPS (1 μg/mL) for 6 hr, followed by treatment with ATP (2.5 mM) for 15 min. (A and B) Effects of NS1-WSN Δ40–80 on IL-1β and IL-18 secretion. The supernatants were harvested and subjected to ELISA to quantify IL-1β and IL-18. Data represent the mean and standard deviation. Statistical analysis was performed using Student’s *t*-test to analyze the differences between control and NS1-expressing samples (*** denotes a *p*-value of <0.005.). (C and D) Effects of NS1-WSN Δ40–80 on NLRP3 inflammasome and NF-κB activation. The cell lysates were obtained and subjected to western blot analysis using the indicated antibodies. (E) Interaction of NS1-WSNΔ40–80 with endogenous NLRP3. Following the abovementioned treatments, the cells were harvested and lysates were immunoprecipitated with anti-MYC antibody. Endogenous NLRP3 protein interaction with NS1 was identified by western blot analysis with anti-NLRP3.
(PDF)
