# Correction: Influenza A Virus NS1 Protein Inhibits the NLRP3 Inflammasome

**DOI:** 10.1371/journal.pone.0200624

**Published:** 2018-07-10

**Authors:** Woo-Chang Chung, Hye-Ri Kang, Hyunyee Yoon, Suk-Jo Kang, Jenny P.-Y. Ting, Moon Jung Song

The first author’s name is spelled incorrectly. The correct name is: Woo-Chang Chung. The correct citation is: Chung W-C, Kang H-R, Yoon H, Kang S-J, Ting JP-Y, Song MJ (2015) Influenza A Virus NS1 Protein Inhibits the NLRP3 Inflammasome. PLoS ONE 10(5): e0126456. https://doi.org/10.1371/journal.pone.0126456
