# Supplementary figures and images for: Influenza A Virus NS1 Protein Inhibits the NLRP3 Inflammasome
Source: PLoS One. 2015 May 15;10(5):e0126456. doi: 10.1371/journal.pone.0126456 (PMC4433236; doi:10.1371/journal.pone.0126456)

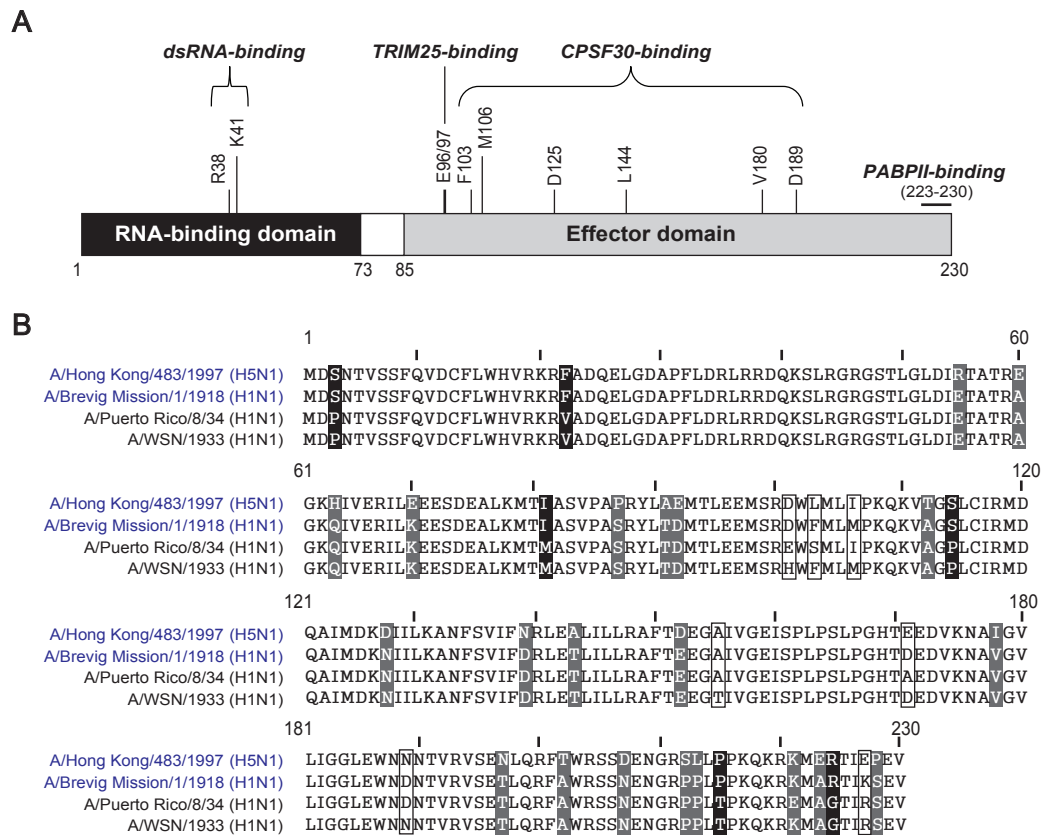

Supplementary figure 1. Cheong *et al.*

Supplement: S1 Fig — (A) A schematic diagram of NS1 protein indicating functional domains and residues critical for its binding with dsRNA, TRIM25, CPSF30, and PABPII. (B) Sequence alignment of NS1 variants. NS1 proteins of highly pathogenic influenza A viruses such as A/Brevig Mission/1/1918 H1N1 and A/Hong Kong/483/1997 H5N1 (blue letters) and those of low pathogenic influenza A viruses such as A/Puerto Rico/8/34 H1N1 and A/WSN/1933 H1N1 (black letters) were aligned by the ‘Clustal Omega’ multiple sequence alignment program. Black boxes indicate six amino acid residues that were conserved among highly pathogenic strains, but not among low pathogenic strains. Gray boxes indicate residues that are different between the H1N1 strains and the H5N1 strain. White boxes indicate residues that are different, but non-characteristic for pathogenic strains. Every tenth amino acids are marked by bars. (PDF) [file pone.0126456.s001.pdf]

**A**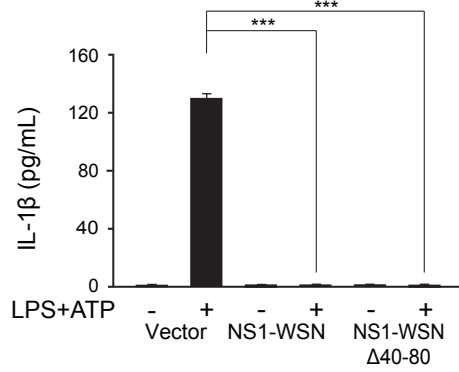**C**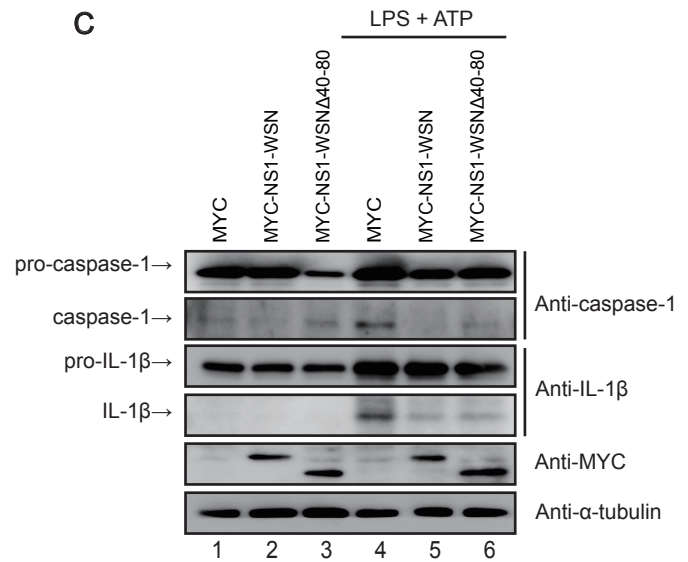**B**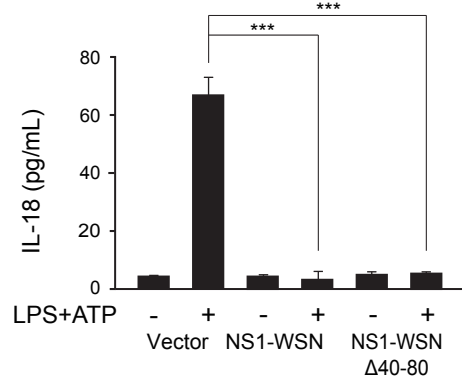**D**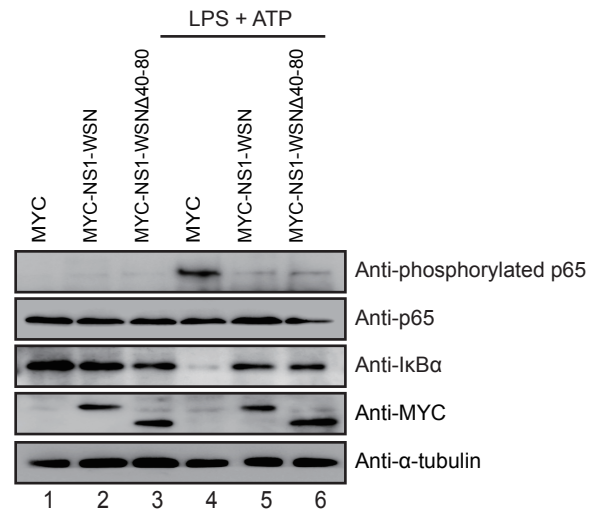**E**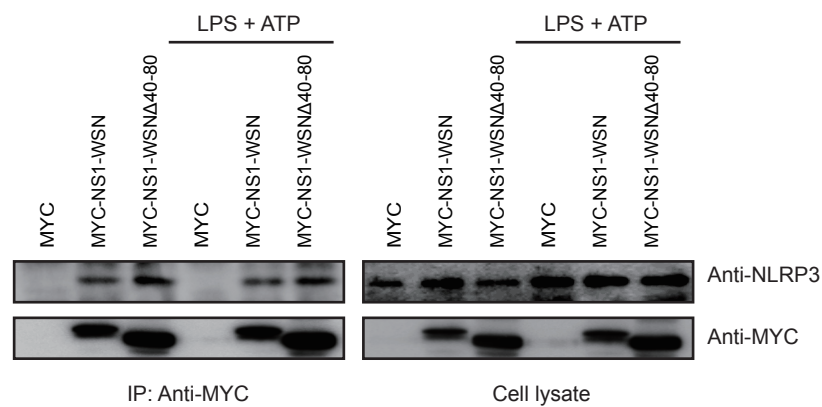

Supplementary figure 2. Cheong *et al.*

Supplement: S2 Fig — THP1 cells were transduced with lentiviruses expressing MYC-tag alone, MYC-NS1-WSN, or MYC-NS1-WSN Δ40–80, and then differentiated with TPA. Differentiated THP-1 cells expressing NS1-WSN or NS1-WSN Δ40–80 were, then, treated with LPS (1 μg/mL) for 6 hr, followed by treatment with ATP (2.5 mM) for 15 min. (A and B) Effects of NS1-WSN Δ40–80 on IL-1β and IL-18 secretion. The supernatants were harvested and subjected to ELISA to quantify IL-1β and IL-18. Data represent the mean and standard deviation. Statistical analysis was performed using Student’s t-test to analyze the differences between control and NS1-expressing samples (*** denotes a p-value of <0.005.). (C and D) Effects of NS1-WSN Δ40–80 on NLRP3 inflammasome and NF-κB activation. The cell lysates were obtained and subjected to western blot analysis using the indicated antibodies. (E) Interaction of NS1-WSNΔ40–80 with endogenous NLRP3. Following the abovementioned treatments, the cells were harvested and lysates were immunoprecipitated with anti-MYC antibody. Endogenous NLRP3 protein interaction with NS1 was identified by western blot analysis with anti-NLRP3. (PDF) [file pone.0126456.s002.pdf]
